# Nano MnO_2_ Radially Grown on Lignin-Based Carbon Fiber by One-Step Solution Reaction for Supercapacitors with High Performance

**DOI:** 10.3390/nano10030594

**Published:** 2020-03-24

**Authors:** Chenyan Guo, Haitong Ma, Qingtong Zhang, Mingfu Li, Hongrui Jiang, Changzhou Chen, Shuangfei Wang, Douyong Min

**Affiliations:** 1 College of Light Industry and Food Engineering, Guangxi University, Nanning 530004, China; guochenyan2017@163.com (C.G.); mahaitong2017@163.com (H.M.); qingyutong110@163.com (Q.Z.); mingfuli@mail.gxu.cn (M.L.); hrjiang@gxu.edu.cn (H.J.); chenchangzhou@gxu.edu.cn (C.C.); wangsf@gxu.edu.cn (S.W.); 2 Guangxi Key Laboratory of Clean Pulp Papermaking and Pollution Control, Nanning 530004, China

**Keywords:** electrospinning, lignin, carbon fiber, mno_2_, supercapacitor

## Abstract

MnO_2_-deposited lignin-based carbon fiber (MnO_2_-LCF) mats are fabricated for supercapacitor applications. LCF mats are produced from alkali lignin via electrospinning followed by stabilization and carbonization. The carbonization process is carried out at 800, 900, and 1000 °C, and the corresponding mats are denoted as MnO_2_-LCF-800, MnO_2_-LCF-900, and MnO_2_-LCF-1000, respectively. The LCF mats are immersed in a KMnO_4_ solution at room temperature for 72 h to obtain MnO_2_-LCF mats. The scanning electron microscopy and X-ray diffraction analysis confirm the deposition of MnO_2_ on the LCFs. The Brunauer–Emmett–Teller analysis, X-ray spectroscopy, and Raman spectroscopy reveal that MnO_2_-LCF-800 mat possesses a large number of mesopores and Mn vacancies as compared to MnO_2_-LCF-900 mat and MnO_2_-LCF-1000 mat. Consequently, MnO_2_-LCF-800 mat possesses the best electrochemical properties with a specific capacitance of 131.28 F∙g^−1^, an energy density of 14.77 Wh∙kg^−1^, and a power density of 135.01 W∙kg^−1^ at a specific current of 0.3 A∙g^−1^. Hence, MnO_2_-LCF-800 mat shows high potential to be used as a high-performance supercapacitor.

## 1. Introduction

With the rapid depletion of energy and the deterioration of the environment, the development of the energy conversion and energy storage technologies has become imperative. Lithium-ion batteries, fuel cells, and supercapacitors have emerged as a new generation of energy storage devices [1,2]. Supercapacitors, which exhibit rapid charging and discharging, high power density, and long cycling life, are a combination of traditional double-layer capacitors (high power output) and fuel cells (high energy storage), and hence find applications in electronic products, automobiles, industrial, and energy management [2,3,4,5,6].

Depending on the storage mechanism, supercapacitors are classified as double-layer capacitors and pseudocapacitors [7,8]. Compared to double-layer capacitors, pseudocapacitors show high energy and power densities. This is because fast and reversible redox reactions occur at or near the electrode surface of pseudocapacitors [9]. Transition metal oxides or hydroxides such as RuO_2_, MnO_2_, Co_3_O_4_, Ni(OH)_2_, and Co(OH)_2_ are the most widely used pseudocapacitive electrode materials [10,11,12,13,14]. Among these oxides, MnO_2_ is the most promising electrode material owing to its low cost, low toxicity, and large theoretical capacity [15]. In aqueous electrolytes, pseudocapacitive charge storage in MnO_2_ occurs through the redox reaction of Mn (+4 and +3 oxidation states) at the surface. MnO_2_ exhibits two charge storage mechanisms [16]. The first mechanism involves the intercalation and de-intercalation of cations in the bulk material, representing the redox reaction of Mn ions. The second mechanism involves the adsorption of electrolyte cations onto the surface of MnO_2_, which involves a change in the oxidation state of Mn from +3 to +4 during the redox reaction [17].

However, MnO_2_ shows low electronic conductivity and electrochemical reaction rate in aqueous electrolytes. An efficient approach to improve the performance of MnO_2_ electrodes is the use of conductive materials such as carbon nanotubes (CNTs) [18], graphene [19], and carbon fibers [20]. Li et al. [18] prepared MnO_2_/CNT composites through a modified one-pot reaction process by coating CNTs with uniformly cross-linked MnO_2_ flakes. The composites exhibited a specific capacitance of 201 F∙g^−1^ at 1 A∙g^−1^ and delivered a high energy density of 13.3 Wh∙kg^−1^ at a power density of 600 W∙kg^−1^. Dai et al. [19] prepared graphene oxide nanosheet/MnO_2_ nanowire composites via a hydrothermal process. The composites showed a specific capacitance of up to 360 F∙g^−1^ at a current density of 0.5 A∙g^−1^ in 1 M Na_2_SO_4_. Hong et al. [20] fabricated a MnO_2_-anchored carbon fiber cloth composite through a simple hydrothermal reaction and used it as a supercapacitor electrode. The composite electrode showed a high specific capacitance of 194 mF∙cm^−2^ at a charge-discharge current density of 2 mA∙cm^−2^ and a high energy density of 0.108 mW∙cm^−2^ at a power density of 2 mW∙cm^−2^. Carbon fiber electrodes are easy to prepare, do not need any binder, and exhibit low resistance.

From the viewpoint of energy conservation and environmental protection, carbon fibers prepared from biomass resources have gained significant attention. Lignin, as a natural raw material, is abundantly available in plant tissues and has a carbon content of more than 60%. Thus, lignin has been extensively investigated as a raw material for preparing carbon fibers. Owing to its simple operation and low cost, electrospinning is widely used to prepare carbon fibers. Moreover, lignin-based carbon fibers (LCFs) prepared via electrospinning are resistant to chemical corrosion and are highly flexible, and hence can be directly used as electrodes without adding the binder. Youe et al. [21] prepared LCFs by mixing lignin and polyacrylonitrile. The MnO_2_/carbon fiber composite showed excellent specific capacitance, energy density, and power density at a specific current. However, the carbon fibers were obtained at the carbonization temperature of 1400 °C. It is well known that the high temperature carbonization requires high quality equipment and consumes enormous energy, which in turn increases the cost of supercapacitors. However, there is little investigations on the LCF prepared at the low carbonization temperature was used to prepare supercapacitors. The effect of LCF properties on the performance of MnO_2_-decorated LCF composite supercapacitor electrodes should be investigated. In this study, environmentally friendly and degradable polyvinyl alcohol (PVA) and lignin were used as the precursors to prepare low carbonization temperature LCFs, which were then used to prepare MnO_2_-loaded LCF composites. The effects of the structure, specific surface area, pore structure, and element valence of the MnO_2_-loaded LCF composites on their electrochemical performance were investigated. The MnO_2_-loaded LCF composites showed great potential to be used as supercapacitor electrodes.

## 2. Experimental

### 2.1. Materials

Alkali lignin powder was purchased from Shanghai Chemicals Industrial Development Company (Shanghai, China). PVA was purchased from Aladdin Company (Shanghai, China). Potassium permanganate (KMnO_4_) and glacial acetic acid were purchased from Guangxi Nanning Rongyi Experimental Equipment Company (Nanning Guangxi, China). All the chemicals were used as-received without further purification.

### 2.2. Preparation of LCFs

The LCFs were prepared from alkali lignin via electrospinning followed by stabilization and carbonization. Briefly, 2 g of PVA was dissolved in 18 g of deionized water with stirring at 80 °C to obtain a PVA solution. Then, 24.5 g of alkali lignin was added to 10.5 g of the PVA solution followed by the addition of 65 g of glacial acetic acid. The resulting mixture was heated at 80 °C with continuous stirring for 4 h. The spinning solutions were loaded into a plastic syringe attached to a 25 G needle in an electrospinning machine (SS-2535H, Yongkang, Beijing, China). The lignin/PVA composite fibers were collected in the form of overlaid mats on an aluminum foil. The electrospinning voltage was 20 kV. The distance between the needle and the collector was 15 cm, feeding rate was 0.5 mL∙h^−1^, and collector drum rotating speed was 150 rpm. The as-spun fiber mats were finally vacuum-dried overnight at 40 °C.

The as-spun mats were transferred to a quartz boat, which was then placed in a tube furnace (T1260A, Chengyi instruments, Henan, China) for stabilization and carbonization. The following temperature program was employed for carrying out the stabilization process. (1) Temperature was increased to 105 °C at 5 °C∙min^−1^ and maintained for 1 h to remove the moisture. (2) The temperature was then increased to 220 °C at 0.5 °C∙min^−1^ and maintained for 20 h for stabilization. The stabilized fiber mat was carbonized as follows. The temperature of the furnace was increased to 800, 900, and 1000 °C at 5 °C∙min^−1^ and maintained for 1 h under a continuous flow of nitrogen to complete the carbonization process. The LCF mats obtained at the carbonization temperatures of 800, 900, and 1000 °C were labeled as LCF-800, LCF-900, and LCF-1000, respectively.

### 2.3. Preparation of MnO_2_-deposited LCF (MnO_2_-LCF) Mats

For preparing the MnO_2_-LCF mats, 2 mg of the LCF mats (LCF-800, LCF-900, and LCF-1000) were immersed in 20 mL of KMnO_4_ (2 mM) at room temperature for 72 h. After the completion of the reaction, the samples were removed from the solution and washed with DI water three times. The samples were then dried at 60 °C for 3 h to obtain the MnO_2_-LCF mats. The mass of MnO_2_ on the LCF surface was determined by the mass discrepancy after the deposition. The MnO_2_-LCF mats prepared using LCF-800, LCF-900, and LCF-1000 were labeled as MnO_2_-LCF-800, MnO_2_-LCF-900, and MnO_2_-LCF-1000, respectively. The mass loading of MnO_2_ in LCF mats were calculated using the following equations:

M = (m_2_ − m_1_)/s (1)

where M is the mass loading of MnO_2_ in LCF mats (mg∙cm^−2^), **m_1_** is the mass of LCF mats before reaction (mg), m**_2_** is the mass of LCF mats after reaction (mg), **s** is the area of mats(cm^2^).

### 2.4. Characterization of LCF and MnO_2_-LCF

The morphologies of the LCF and MnO_2_-LCF samples were examined using scanning electron microscopy (SU8220, Hitachi, Japan). The structures of the samples were investigated using X-ray diffraction (XRD) (MiniFlex 600, Rigaku Corporation, Japan) and Raman spectroscopy (LabRAM HR Evolution, HORIBA Jobin Yvon, France). The XRD measurements were carried out over the 2θ range of 10–80° at the scanning rate of 5°∙min^−1^. The surface elemental compositions of the samples were analyzed using X-ray photoelectron spectroscopy (ESCALAB 250Xi, Thermo Fisher Scientific, Waltham, MA, USA). The specific surface areas and pore sizes of the samples were determined by Brunauer–Emmett–Teller (BET) analysis (ASAP 2460, Micromeritics, Atlanta, GA, USA).

### 2.5. Electrochemical Performance of MnO_2_-LCF

The electrochemical performances of the MnO_2_-LCF mats were investigated by cyclic voltammetry (CV), galvanostatic charge/discharge (GCD) measurements, and electrochemical impedance spectroscopy (EIS). The electrochemical measurements were carried out on an electrochemical workstation (MULTI AUTOLAB M204, Metrohm, Switzerland). The MnO_2_-LCF mats were cut into specimens with the dimensions of 1 × 1 cm. The mass of MnO_2_-LCF-800, MnO_2_-LCF-900, and MnO_2_-LCF-1000 were 2.74 mg, 3.70 mg, and 3.58 mg, respectively. Then the specimens were sandwiched between two nickel foams. The sandwich was then pressed at 10 MPa to form the electrode using a tablet press (FW-4A, Tianguang instruments, Tianjin, China). A three-electrode system with a platinum sheet electrode as the counter electrode, a saturated calomel electrode as the reference electrode, and 1M NaSO_4_ as the electrolyte was used. The CV measurements were carried out at various scan rates (5–100 mV∙s^−1^) over the potential range of 0–0.9 V. The GCD measurements were carried out over the current density range of 0.3–1 A∙g^−1^. The EIS measurements were carried out over the frequency range of 0.01 Hz–100 kHz at an amplitude of 20 mV. The specific capacitances, energy densities, and power densities of the samples were calculated using the following equations [22].

C=(I∙t)∙(U∙m)^−1^ (2)

E=0.5∙CU^2^ (3)

P=E/t(4)

where C denotes the specific capacitance (F∙g^−1^), E is the energy density (Wh∙kg^−1^), P is the power density (W∙kg^−1^), I is the discharge current (A), t is the discharge time (s), U is the voltage change (V), and m is the mass of MnO_2_-LCF in the electrode (g).

## 3. Results and Discussion

### 3.1. Effect of Carbonization Temperature on the Morphology and Structure of the LCF Mats

**Figure 1:**
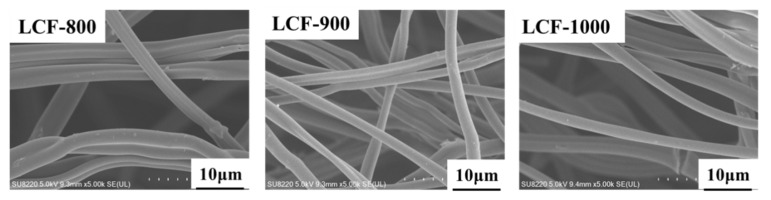
Surface morphologies of the lignin-based carbon fiber (LCF) mats prepared at different carbonation temperatures.

As can be observed from Figure 1, the LCF mats showed smooth and uniform surfaces. The average diameters of LCF-800, LCF-900, and LCF-1000 were 1.52, 1.09, and 0.92 μm, respectively. This indicates that the diameter of the LCFs decreased with an increase in the carbonization temperature. This can be attributed to the mass loss caused by the loss of non-carbon elements from the LCFs in the form of volatiles at high carbonization temperatures [23,24].

**Figure 2:**
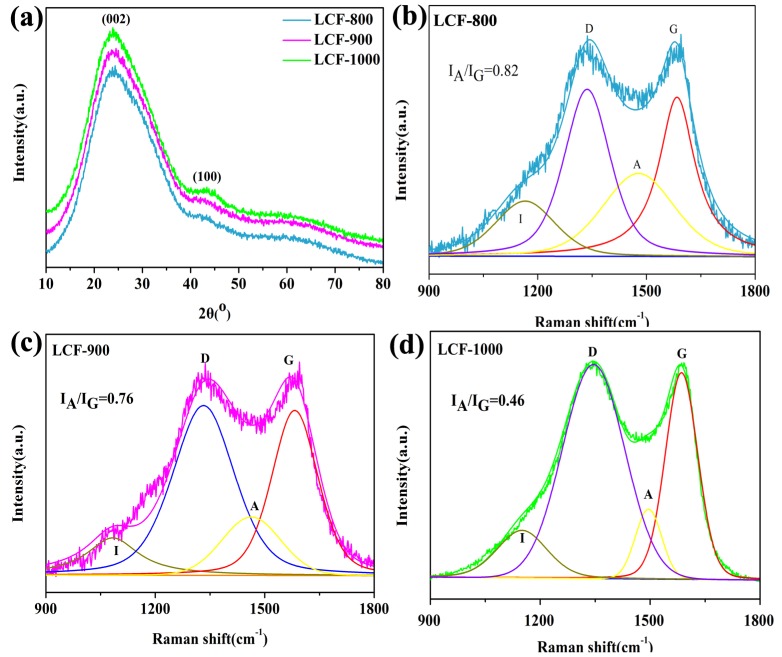
XRD spectra of the LCF mats (**a**); Raman spectra of LCF-800 (**b**), LCF-900 (**c**), and LCF-1000 (**d**).

As can be observed from Figure 2a, all the samples showed two broad diffraction peaks at 24 and 44° corresponding to the (002) plane of graphitic carbon and the (100) plane of amorphous carbon, respectively. This indicates that the LCF mats showed a highly amorphous structure. These two peaks confirm the conversion of lignin into carbonaceous materials [25]. The deconvoluted Raman spectrum of LCFs shows an impurity band (I) at 1140 cm^−1^, a disordered graphitic band (D) at 1348 cm^−1^, an amorphous band (A) at 1484 cm^−1^, and an ordered graphitic band (G) at 1585 cm^−1^ [26,27]. The A/G band intensity ratio of carbon materials is a measure of their amorphous degree [28]. The I_A_/I_G_ values of LCF-800, LCF-900, and LCF−1000 were 0.82, 0.76, and 0.46, respectively, indicating that the amorphous degree of LCF-800 was higher than those of LCF-900 and LCF-1000. This is consistent with the results reported by Chatterjee et al. [29]. These results suggest that the carbonization temperature is the key factor affecting the morphology and structures of LCFs. The average diameter and amorphous degree of the LCFs decreased with an increase in the carbonization temperature. LCFs with high amorphous degrees provide significantly larger transport channels for electrolyte ions than those with graphitized structures. These transport channels increase the rate of ion diffusion through the electrode [30]. 

### 3.2. Morphology and Structure of MnO_2_-LCF

**Figure 3:**
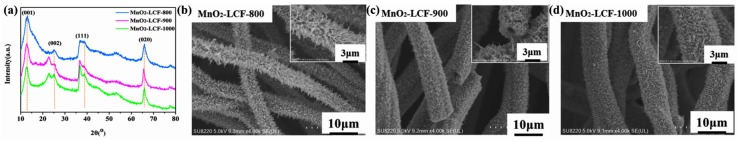
XRD patterns of the MnO_2_-LCF mats (**a**); SEM images of the MnO2-LCF-800 (**b**), MnO_2_-LCF-900 (**c**), MnO_2_-LCF-1000 mats (**d**).

From Figure 3a, it can be observed that the diffraction peak positions of all the samples were identical. The diffraction peaks at 12.5, 25, 37, and 65° correspond to the (001), (002), (100), and (110) planes of Birnessite-type MnO_2_ (JCPDS No. 80-1098), respectively. The core-shell structure of the sample is revealed in Figure 3b–d. The core layer was composed by LCF, and the shell layer was composed by MnO_2_. After the deposition of MnO_2_ on the surface, the average diameter of LCF-800 increased from 1.52 to 2.37 μm. The average diameters of LCF-900 increased from 1.09 to 3.56 μm. The average diameter of LCF-1000 increased from 0.92 to 4.18 μm. Therefore, the thickness of the MnO_2_ layer was calculated as 0.43, 1.24, and 1.63μm, respectively. In addition, the mass loading of nO_2_ on LCF-800, LCF-900, and LCF-1000 was calculated (using Equation (1)) as 0.69, 2.21, and 2.39 mg∙cm^−2^, respectively. It has been reported that the specific capacitance decreased with the increase of MnO_2_ loading [31].

**Figure 4:**
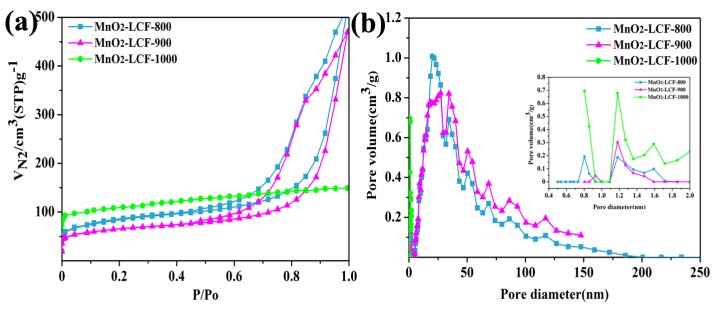
N_2_ adsorption/desorption isotherms of the MnO_2_-LCF samples (**a**) and the corresponding BJH pore size distributions (**b**).

Figure 4a shows the N_2_ adsorption-desorption isotherms of the MnO_2_-LCF samples. Both MnO_2_-LCF-800 and MnO_2_-LCF-900 showed type-II isotherms. When the relative pressure was close to the saturated vapor pressure, the equilibrium state could not be reached, indicating that MnO_2_-LCF-800 and MnO_2_-LCF-900 consisted mainly of mesopores [32]. On the other hand, MnO_2_-LCF-1000 showed a type-I N_2_ adsorption/desorption isotherm, indicating that it mainly consisted of micropores [33]. This is why the specific surface area of MnO_2_-LCF-1000 was larger than those of MnO_2_-LCF-800 and MnO_2_-LCF-900 (Table 1).

The BJH (Barrett, Joyner and Halenda) method was used to investigate the pore size distributions (using the desorption curve) of the MnO_2_-LCF samples. Figure 4(b) shows that MnO_2_-LCF-800 and MnO_2_-LCF-900 consisted mainly of mesopores (2–50 nm). On the other hand, MnO_2_-LCF-1000 was microporous (0.8–2 nm). The pores of MnO_2_-LCF-800 were larger than those of MnO_2_-LCF-900 and MnO_2_-LCF-1000. These results indicate that MnO_2_-LCF-1000 was not suitable to be used as a supercapacitor electrode because its micropores limited its ion diffusion rate [34]. On the other hand, MnO_2_-LCF-800 was found to be suitable for use as a supercapacitor electrode. This is because its mesopores provided a low resistance channel and short diffusion path for the diffusion of ions [34].

**Figure 5:**
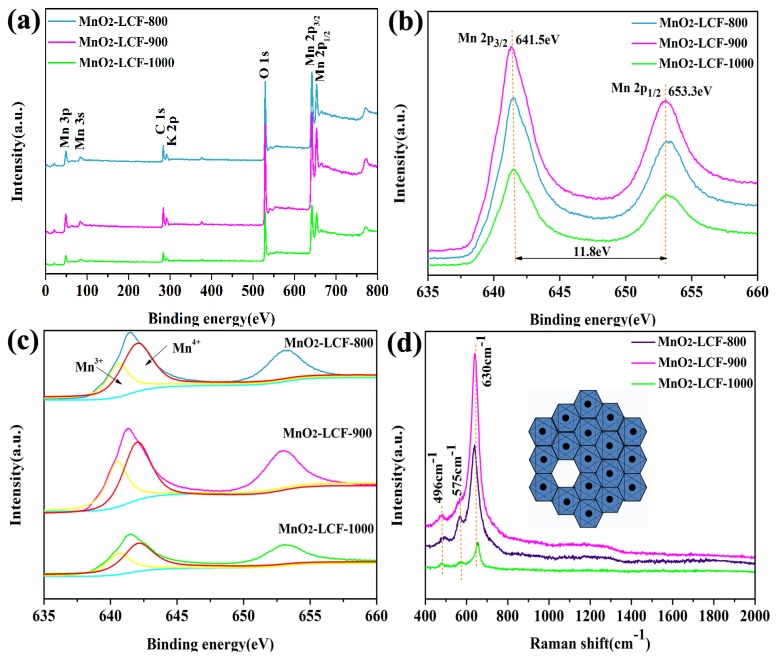
XPS spectra of the MnO_2_-LCF samples (**a**); Mn2p spectra (**b**); fitting results of Mn2p_3/2_ spectra (**c**); Raman spectra of the MnO_2_-LCF mats (**d**). The built-in illustration is the top view of Birnessite-type MnO_2_ along the direction of [001], and the vacancy in the picture is the vacancies in MnO_2_.

From Figure 5a, it can be observed that the MnO_2_-LCF mats consisted of C, O, Mn, and K. The appearance of the carbon peaks can be attributed to the incomplete reaction of KMnO_4_ with the LCFs. In addition, Figure 5b shows that Mn2p_3/2_ and Mn2p_1/2_ peaks were observed at 641.5 and 653.3eV, respectively, and the spin energy difference was 11.8 eV. Indicating that the average oxidation state of the MnO_2_ whiskers in the MnO_2_-LCF mats was mainly +4 [35]. However, the Mn2p_3/2_ spectra (Figure 5c) revealed the presence of both Mn^3+^ and Mn^4+^ [36]. The Mn^3+^ proportions of MnO_2_-LCF-800, MnO_2_-LCF-900, and MnO_2_-LCF-1000 were 36.84%, 38.46%, and 40.74%, respectively. Hence, MnO_2_-LCF-800 showed the lowest Mn^3+^ content among all the samples. Mn^3+^ can reduce the number of vacancies in MnO_2_, thus limiting the storage and release of charges during the charging and discharging process because electrolyte cations cannot enter the bulk phase of MnO_2_ through the surface of the electrode to complete the embedding and removal process [37,38].

MnO_2_-LCF-800 showed the strongest Raman peak at 575 cm^−1^ (characteristic peak of Birnessite-type MnO_2_) (Figure 5d), suggesting that it showed the highest Mn^4+^ and vacancy contents among all the samples [39]. This is consistent with the XPS results, which showed that the Mn/O ratios of MnO_2_-LCF-800, MnO_2_-LCF-900, and MnO_2_-LCF-1000 were 0.74, 0.76, and 0.78, respectively, indicating MnO_2_-LCF-800 showed more vacancies than MnO_2_-LCF-900 and MnO_2_-LCF-1000 (Table 2). Therefore, compared to MnO_2_-LCF-900 and MnO_2_-LCF-1000, MnO_2_-LCF-800 was found to be more suitable to be used as an electrode material.

### 3.3. Electrochemical Performance of MnO_2_-LCF

**Figure 6:**
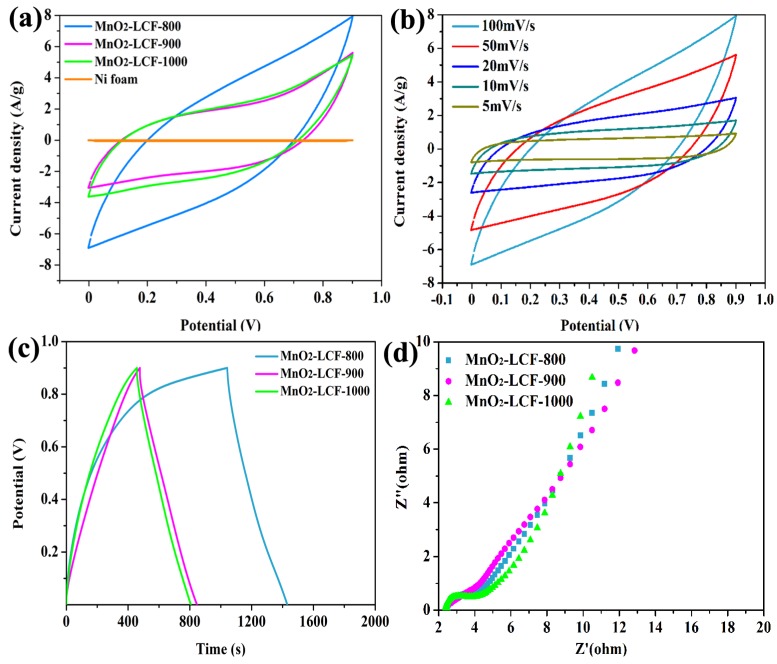
CV curves of the MnO_2_-LCF mat electrodes at a constant scan rate of 100 mV∙s^−1^ within the potential range of 0–0.9 V (**a**), CV curves of the MnO_2_-LCF-800 mat electrode at different scan rates within the potential range of 0–0.9 V (**b**), GCD curves the MnO_2_-LCF mat electrodes at the current density of 0.3 A∙g^−1^ (**c**), Nyquist plots of the MnO_2_-LCF mat electrodes (**d**).

The electrochemical performances of the MnO_2_-LCF electrodes were evaluated by carrying out their CV, GCD, and EIS measurements. As can be observed from Figure 6a, the CV curves of the electrodes were near-rectangular without distinguishable redox peaks. This indicates that the MnO_2_-LCF electrodes exhibited an ideal capacitive behavior and high reversibility [40]. At the same scanning rate, MnO_2_-LCF-800 showed larger ring region than MnO_2_-LCF-900 and MnO_2_-LCF-1000, indicating that the MnO_2_-LCF-800 electrode showed the highest capacitance among all the electrodes. In addition, the CV curves of MnO_2_-LCF-800 retained their rectangular shape even at high voltage sweep rates (Figure 6b). This suggests that the MnO_2_-LCF-800 electrode retained its pseudocapacitive behavior even at high voltage sweep rates [41]. 

Interestingly, the shape of the GCD curves of the MnO_2_-LCF electrodes was close to that of an isosceles triangle (Figure 6c). This indicates that the MnO_2_-LCF electrodes showed an ideal charge/discharge behavior, reversibility, good storage capacity, and pseudocapacitance [42]. The specific capacitance, specific power, and specific energy of the MnO_2_-LCF-800 electrode were calculated (using Equations (2), (3), and (4)) to be 131.28 F∙g^−1^, 14.77 Wh∙kg^−1^, and 135.01 W∙kg^−1^, respectively. These values were higher than those of the MnO_2_-LCF-900 and MnO_2_-LCF-1000 electrodes. These results suggest that the MnO_2_-LCF-800 electrode exhibited good charge/discharge depth and storage capacity. 

The Nyquist plots of MnO_2_-LCF-800 and MnO_2_-LCF-900 (Figure 6d) showed semi-circular curves in the high frequency region, indicating light electrochemical polarization. The semicircle radius (representing the magnitude of the charge transfer rate) of MnO_2_-LCF-800 was smaller than that of MnO_2_-LCF-900. On the other hand, the linear slope (representing the diffusion resistance of the electrolyte) of MnO_2_-LCF-800 was larger than that of MnO_2_-LCF-900. This indicates that MnO_2_-LCF-800 showed high ion transport speed and low electrolyte diffusion resistance because of the presence of a large number of mesopores in it. Thus, MnO_2_-LCF-800 was found to be more suitable than the other electrodes for the diffusion of high-speed ions at high load current densities, which is beneficial for the rapid charge transfer between the electrode and the electrolyte [34,43]. Therefore, MnO_2_-LCF-800 showed huge potential to be used as a supercapacitor electrode.

## 4. Conclusions

In this study, nano MnO_2_ flakes radially grown on LCF to obtain MnO_2_-LCF mats, which can be used as freestanding binder-free supercapacitor electrodes. The MnO_2_-LCF-800 electrode exhibited a specific capacitance of 131.28 F∙g^−1^ at 0.3 A∙g^−1^ which was attributed to the optimum mass loading of MnO_2_ on the surface of LCF and the presence of mesopores and vacancies in MnO_2_. The results showed that LCFs obtained at low carbonization temperatures can be modified with MnO_2_ to prepare supercapacitors with excellent performance. These results provide a theoretical basis for the development of low-cost, green, and high-performance supercapacitors.

## Figures and Tables

**Table 1:** Brunauer–Emmett–Teller (BET) analysis of the MnO_2_-LCF samples.

Sample	S_BET_ (m^2^∙g^−1^)	V_mic_ (cm^3^∙g^−1^)	V_t_ (cm^3^∙g^−1^)	V_mic_/V_t_
MnO_2_-LCF-800	273.70	0.03	0.09	33.33
MnO_2_-LCF-900	210.91	0.04	0.07	57.14
MnO_2_-LCF-1000	385.16	0.08	0.12	66.67

Note: S_BET_: the specific surface area; V_mic_: micropore volume; V_t_: total pore volume.

**Table 2:** Mn, O, and C concentrations of the MnO_2_-LCF samples, as determined from their XPS spectra.

Sample	Atomic Percentage of C (%)	Atomic Percentage of Mn (%)	Atomic Percentage of O (%)
MnO_2_-LCF-800	54.54	19.30	26.16
MnO_2_-LCF-900	51.61	19.52	25.62
MnO_2_-LCF-1000	54.54	19.84	25.61
